# Use of structure-activity landscape index curves and curve integrals to evaluate the performance of multiple machine learning prediction models

**DOI:** 10.1186/1758-2946-3-7

**Published:** 2011-02-07

**Authors:** Norman C LeDonne, Kevin Rissolo, James Bulgarelli, Leonard Tini

**Affiliations:** 1 Discovery DMPK, AstraZeneca Pharmaceuticals, Wilmington, Delaware 19850, USA

## Abstract

**Background:**

Standard approaches to address the performance of predictive models that used common statistical measurements for the entire data set provide an overview of the average performance of the models across the entire predictive space, but give little insight into applicability of the model across the prediction space. Guha and Van Drie recently proposed the use of structure-activity landscape index (SALI) curves via the SALI curve integral (SCI) as a means to map the predictive power of computational models within the predictive space. This approach evaluates model performance by assessing the accuracy of pairwise predictions, comparing compound pairs in a manner similar to that done by medicinal chemists.

**Results:**

The SALI approach was used to evaluate the performance of continuous prediction models for MDR1-MDCK *in vitro *efflux potential. Efflux models were built with ADMET Predictor neural net, support vector machine, kernel partial least squares, and multiple linear regression engines, as well as SIMCA-P+ partial least squares, and random forest from Pipeline Pilot as implemented by AstraZeneca, using molecular descriptors from *SimulationsPlus *and AstraZeneca.

**Conclusion:**

The results indicate that the choice of training sets used to build the prediction models is of great importance in the resulting model quality and that the SCI values calculated for these models were very similar to their Kendall τ values, leading to our suggestion of an approach to use this SALI/SCI paradigm to evaluate predictive model performance that will allow more informed decisions regarding model utility. The use of SALI graphs and curves provides an additional level of quality assessment for predictive models.

## 1. Background

The use of biological property predictions has increased in recent years, due to improvements in computer technology, the rising costs of drug discovery, and a desire by regulatory agencies to better understand, predict and improve drug safety [1-7]. The increase in computer processing speed has allowed very complex and computationally intensive models to be developed and run on desktop computers. Model building is becoming more of a routine practice, and will likely continue to grow in importance. However, the pace of proliferation of models and model building tools has not been matched by development of approaches and tools to rigorously assess their performance.

In a recent set of papers, Guha and Van Drie have proposed the Structure Activity Landscape Index (SALI) [8,9] as an approach to better assess biochemical structure-activity relationship (SAR) model performance. The concept is derived from the observation that activities based on specific interactions (as in receptor binding) do not change linearly with linear changes in properties. For example, while building an SAR, increasing the length of an alkyl substituent may result in a 0.3 log increase in potency per carbon, when one to three carbons are added; however, addition of the fourth carbon may increase the potency by 1 log unit, constituting what Guha and Van Drie have referred to as an "activity cliff". By identifying these activity cliffs within an SAR set the SALI procedure can improve the understanding of where the model is more or less accurate.

Quantification of the structure activity cliffs is carried out by pairwise comparisons of compounds and their related measured and predicted activities, an approach similar to that routinely taken by medicinal chemists in the generation of SAR. In practice, the activity differentials are normalized by their structural similarity measures (for example, Tanimoto similarities). A small SALI value is indicative of a smooth activity transition, whereas a large SALI value indicates the presence of an activity cliff. The SALI graph of the dataset is a representation of its SAR as a connected graph, with molecules as the nodes and the SALI_ij _values as edges. Plotting the sum of the nodes normalized by the edges versus the normalized threshold for edge detection generates a SALI curve. The value of the curve at X = 0 (S(0)) provides the ability of the model to capture all of the edges while the value at X = 1 (S(1)) is the ability of the model to correctly identify the most significant activity cliffs.

Because of the recognized importance of transporters in drug absorption, distribution and elimination [10,11]*in vitro *and *in silico *models to predict transporter involvement have been established. The MDCK-MDR1 *in vitro *model uses an immortalized mammalian cell line that stably expresses the transfected human MDR1 gene product (P-glycoprotein (P-gp)) to assess the potential for P-gp mediated efflux of compounds.

MDR1 efflux is dependent on specific structural features of the transported molecule, a key requirement for the application of the SALI/SCI approach. The general features of this interaction have been described by Anna Seelig [12]. Seelig determined that the likelihood of a molecule to bind to and to be an efflux substrate is based on the presence and the intermolecular distance between 2 hydrogen bond acceptors. Theoretically, incorporation of these 3D descriptors should increase the accuracy of efflux predictions.

*SimulationsPlus *ADMET Predictor software enables models to be built using neural networks (ANNE), support vector machine (SVM), multiple linear regression (MLR) and Kernel Partial Least Squares (KPLS) approaches. In addition, *Umetrics *SIMCA-P+ provides a partial least squares (PLS) prediction using principal component analysis (PCA). Finally, AstraZeneca has implemented the *Accelrys *Pipeline Pilot random forest (RF) model builder.

The results of SALI, S(0), S(1), Kendall τ, and MAE assessments of various model building approaches for their utility in predicting MDR1 mediated efflux as measured in an *in vitro *assay using a training set derived from the same *in vitro *data set are compared here.

## 2. Experimental

### Datasets

MDR1-MDCK efflux data were generated from compounds synthesized and tested in support of drug discovery projects at AstraZeneca (Wilmington DE) using an MDCK-MDR1 transwell assay. All chemicals used in the assay were of at least reagent grade. The assay was conducted using MDR1-MDCK cells seeded at a density of 60,000 cells/well in DMEM medium with Glutamax into Millipore 96 well plates, to final volumes of 100 μL on the apical side and 310 μL on the basolateral side. Cells were grown for 3 to 5 days at 37°C in a humidified 5% CO2 atmosphere, with daily medium changes including a final medium change two hours prior to running the assay. At the initiation of the efflux assay, the media on both the apical and the basolateral sides was replaced with the same volume of warmed Hank's Balanced Salts Solution with or without 1 μM test compound. The cells were incubated at 37°C for 2.5 h, and then the concentrations of compound in the apical and basolateral side were quantitated by LC/MS/MS using standard analytical methods. Cell layers were tested for integrity by addition of 100 μM Lucifer Yellow in Hank's Balanced Salts Solution to the transwell chamber corresponding to the apical side of the cell monolayer. The fluorescence in the basolateral chamber was quantified after incubation for 1 hour at 37°C. Only data from wells with Lucifer Yellow readings demonstrating less than 0.5% cell leakage were reported. Experimental runs were accepted based on the performance of standards with known efflux ratios.

**Figure 1:**
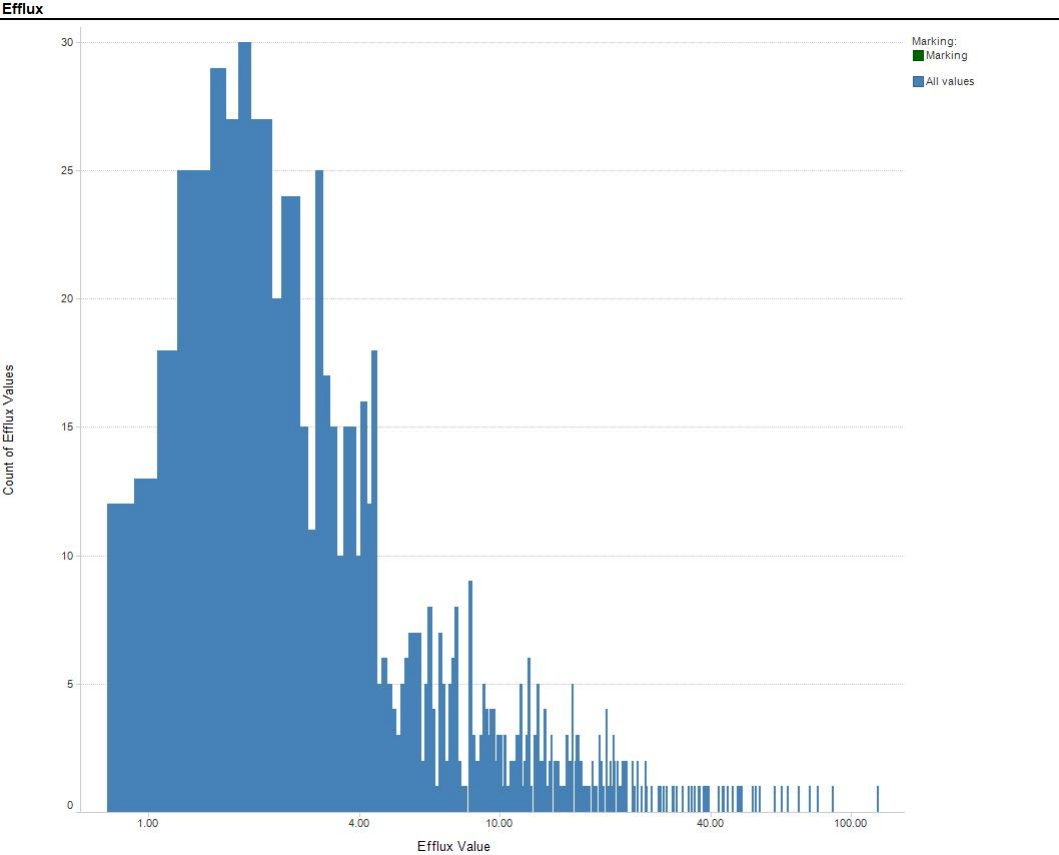
**Distribution of Efflux Values used for Training Prediction Models**. Count of SALI values per bin versus SALI value

The following criteria were used to select the compounds in the training and test set: successful generation of SMILES by the SMILES generation algorithm; absence of non-organic elements (a prerequisite of the ADMET Predictor); efflux ratio ≥0.7; coefficient of variation for replicates ≤50% and elimination of censored data. Prediction models were initially generated using ANNE and RF. The structures of the compounds and measured efflux values of outliers from both models were subsequently examined for potential inconsistencies; those compounds with suspect data based on assay irregularities were then removed from the data set. The outcome of this process was a set of 818 compounds with efflux ratios between 0.7 and 119. The distribution of the efflux values in the final dataset is shown in Figure 1.

The dataset was separated into training and test sets using two different approaches. The *ADMET Predictor *software uses Kohonen mapping [13,14] to assign compounds to training and test sets. *ADMET Predictor *uses the same descriptors for generation of the Kohonen map and for the model building. Alternatively, compounds were assigned random numbers between 0 and 1 using the Excel "RAND" function. The data were sorted descending on the random numbers; the top thirty percent (245) of compounds were selected as test compounds, while the remaining 513 compounds were assigned as the training set. In this case, the maximum and minimum measured efflux values were included in the training set to eliminate prediction mod el extrapolation.

To test the different modeling approaches, models were built using the same training/test set data. A Kohonen map was constructed and used for all 2D or 3D models. The file listing the Kohonen map of the compounds was opened using Excel, and the listing of compounds along with their status as training/verify/test was used to define the training (Kohonen training/verify) and test (Kohonen test) values for the RF and PLS models.

### Model Building

#### 2D descriptors

Using ADMET Predictor descriptors and Kohonen map, ANNE, SVM, MLR, KPLS, RF and PLS models were built. In all cases, the same compounds (based on the Kohonen map) were used for test and training/(verify) sets. The ADMET descriptors (325) consisted of molecular weight, number of rings (total, aromatic, aliphatic), numbers of specific functional groups, geometric descriptors (moments of inertia, radii of gyration, surface areas), atomic partial charges, number of heteroatoms, fraction of bonds (single, double, triple), charge, hydrogen bonding descriptors, molecular ionization. The ADMET descriptors did not contain Formula, N_Nonorgn, N_Metal, N_Kekule, S_unknown, Unknown_, AcidAtoms, or BaseAtoms, and therefore these descriptors were not included in the descriptors used for RF and PLS models. The *ADMET Predictor *application automatically removed highly-correlated descriptors, resulting in a final descriptor set of 134 descriptors actually used in ADMET model building. Feature selection was manually performed in by PCA analysis for SIMCA P+ PLS models.

Alternatively, a set of 196 molecular and electronic descriptors utilized within AstraZeneca (AZ descriptors, ANNE AZ) were used. These descriptors consisted of lipophilicity, hydrogen bonding, size and shape, charge, polarity, atom counts, topology and druggability. The ADMET Predictor model building software automatically performed feature selection, resulting in a final descriptor set of 76 descriptors. Feature selection was manually performed in by PCA analysis for SIMCA P+ PLS models.

#### 3D descriptors

Using 3D sd files mgenerated by VIDA3 (OpenEye), 3D descriptors were calculated using *ADMET Predictor*. The notable additional 3D descriptors were principal moments of inertia, second order static moments, solvent accessible areas, and those described by Seelig for identification of compounds that interact with the MDR1 transporter. Seelig's descriptors are: Type I units containing two electron donor groups with a spatial separation of 2.5 ± 0.3 Å or Type II units consisting of two electron donor groups with a spatial separation of 4.5 ± 0.6 Å or three electron donor groups with a spatial separation of 4.5 ± 0.6 Å between the outer groups. After feature selection, a descriptor set consisting of 149 descriptors was used to build models in ADMET Predictor; all descriptors were used for RF and PLS. Feature selection was manually performed in by PCA analysis for SIMCA P+ PLS models.

### Prospective model verification

To more fully evaluate the performance of the models, a set of 96 compounds was tested prospectively in the MDR1-MDCK efflux assay. This set consisted of AZ compounds that were not in the projects used for the training/test set and which met the same selection criteria as described above for the test/training compounds.

The 96 compounds were evaluated using the both 2D and the 3D descriptor sets. All models, with the exception of the random forest model, provided an assessment as to when compounds did not fall within the prediction space of the model. Only those compounds that fell within the prediction space of the respective prediction model were used for the final evaluations of model performance. The final number of compounds varied from 80 to 93, depending on the prediction model. The Pipeline Pilot RF model as implemented at AstraZeneca does not provide a flag for results not within the prediction space of the model; therefore, the RF model was not tabulated in the final analysis of the prospective data set.

After predictions, the parameters MAE, Kendall τ, SCI, S(0) and S(1) were calculated as described below.

## 3. Methods

Mean Absolute Error (MAE) and Kendall τ were calculated using JMP 7. SALI curves and SCI values were calculated as described in Guha and Van Drie [8,9], using Daylight Type Fingerprints to generate Tanimoto similarity scores, and Excel VBA programming to generate the SALI graphs, SCI and S(X) values and SALI curves.

## 4. Results and Discussion

### Comparison of ADMET Predictor, in house RF and SIMCA PLUS PLS using 2D descriptors

#### Training sets

**Figure 2:**
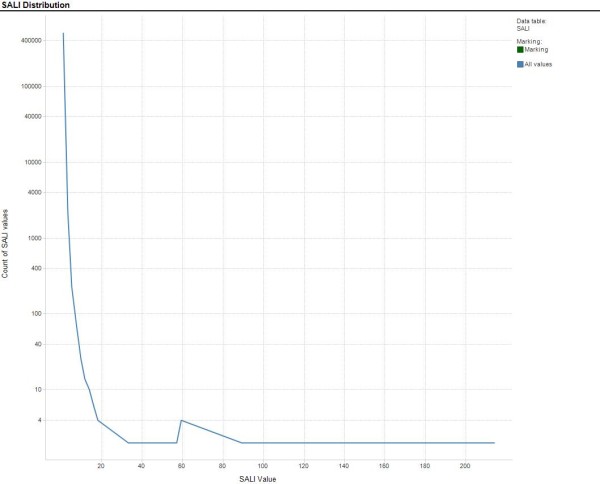
**Distribution of SALI Values Calculated for 2D Training Sets**. Count of efflux value per bin versus efflux value

The distribution of the calculated SALI values are shown in Figure 2. All models with the exception of RF had similar MAE, Kendall τ and S(0) values (Table 1). The results from the RF model suggested that both its accuracy (MAE) and rank ordering (Kendall τ, S(0), SCI) properties were better than the other models tested. Using SCI as an indicator of model performance revealed marked differences between the models. MLR and RF had high SCI values approaching 1, suggesting that they could accurately rank order compounds across most of the edges of the SALI graph. ANNE, SVM, KPLS, PLS and ANNE AZ had SCI values between 0.1 and 0.5, indicating that these models would predict the edges accurately mo re often than inaccurately. Notably, ANNE with the random training/test set had a negative SCI value suggesting that it mispredicted more frequently than it predicted correctly. All S(1) values, except that for ANNE Random, were 1, indicating that all of the models were able to correctly rank order compounds with large SALI values (that is large activity changes with small structural changes) except ANNE Random.

**Table 1:** Summary of 2D model performance

Model	ANNE	SVM	MLR	KPLS	RF	PLS	ANNE AZ	ANNE Random
**Training set**								

MAE	0.19	0.22	0.20	0.19	0.08	0.22	0.20	0.19

Kendall τ	0.63	0.58	0.61	0.63	0.86	0.60	0.60	0.62

SCI	0.12	0.20	0.90	0.48	0.94	0.12	0.17	-0.13

S(0)	0.63	0.57	0.60	0.62	0.86	0.58	0.59	0.62

S(1)	1.00	1.00	1.00	1.00	1.00	1.00	1.00	-1.00


**Test Set**								

MAE	0.22	0.25	0.23	0.24	0.22	0.25	0.21	0.22

Kendall τ	0.51	0.45	0.48	0.48	0.51	0.45	0.53	0.56

SCI	0.83	0.93	0.93	0.94	0.94	0.75	0.96	-0.67

S(0)	0.52	0.46	0.50	0.50	0.52	0.46	0.54	0.56

S(1)	1.00	1.00	1.00	1.00	1.00	1.00	1.00	-1.00


**Prospective Set**								

MAE	0.36	0.32	0.52	0.19	*	0.33	0.36	0.35

Kendall τ	0.34	0.36	0.14	0.37	*	0.32	0.36	0.33

SCI	0.98	0.98	0.72	0.78	*	0.97	0.77	0.98

S(0)	0.35	0.37	0.15	0.38	*	0.33	0.37	0.35

S(1)	1.00	1.00	1.00	1.00	*	1.00	1.00	1.00

Models using ADMET 2D Predictor descriptors and Kohonen map: ANNE, ADMET Predictor neural net; SVM, ADMET Predictor support vector machine; MLR, ADMET Predictor multiple linear regression; KPLS, ADMET Predictor kernel partial least squares; RF, Pipeline Pilot random forest; PLS, SIMCA-P+ partial least squares; ANNE AZ, ADMET Predictor neural net with AZ descriptors; ANNE Random, ADMET Predictor neural net with randomized choice of training/test sets. The performance properties of the models were calculated as described in CALCULATIONS AND STATISTICS. The properties were not calculated for RF since prediction outliers could not be identified.

**Figure 3:**
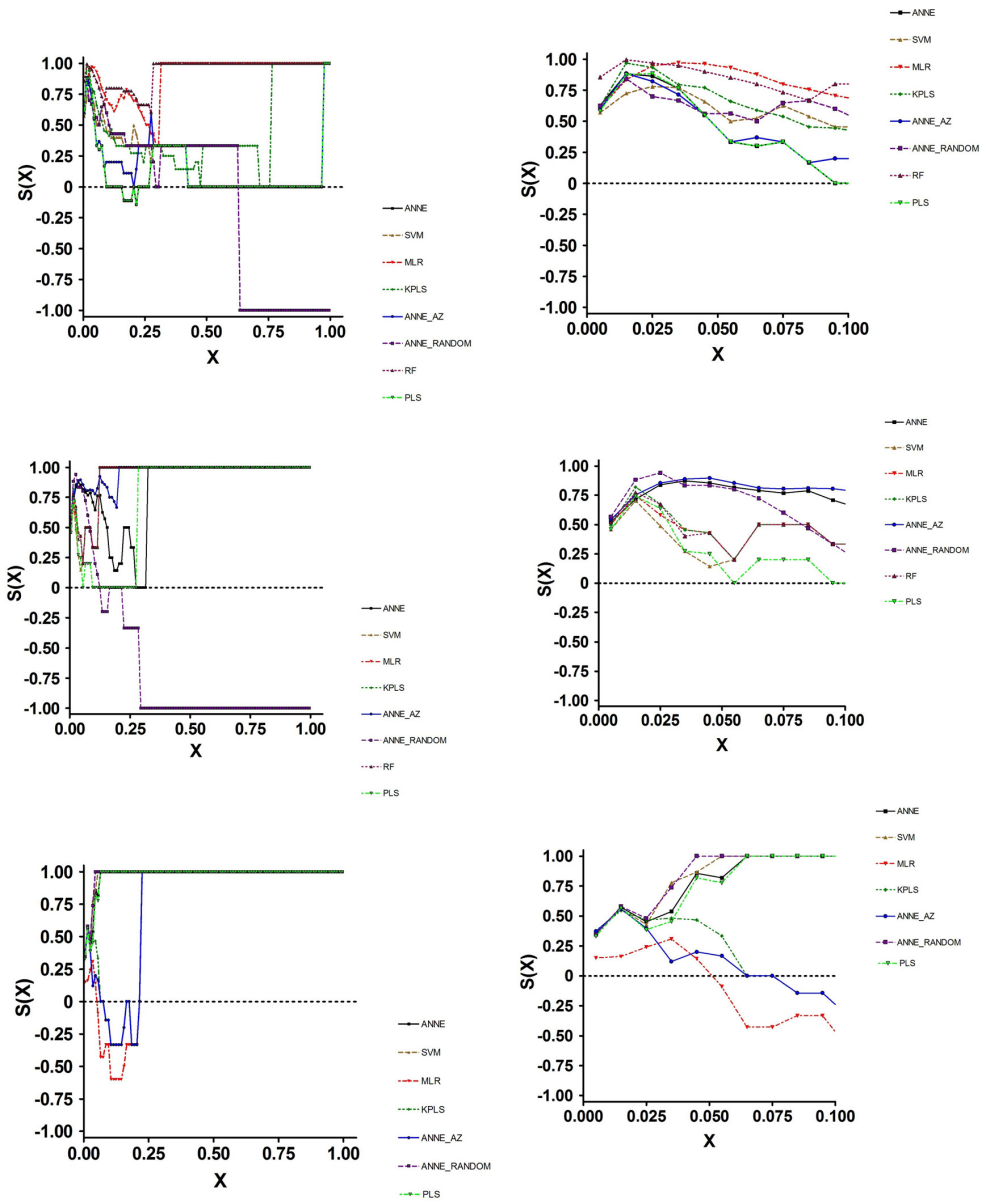
**SALI Curves for Efflux Prediction Data Generated using 2D Descriptors**. 1a, Training Set; 1b, Test Set; 1c, Prospective Set.

Examination of the SALI curves clarified the reason for these results (Figure 3a). All models performed similarly at X < ~0.1. Thus, in those instances where changes in structure resulted in activity changes of a proportional magnitude, all of the models performed equally well. At X values greater than 0.3, MLR and RF could accurately order compound pairs across edges. The other models performed less well. Importantly, at X = 0.65, the S(X) for ANNE Random became negative, indicating that this model performed poorly at larger X values, completely mispredicting the nodes.

#### Test sets

The test set results, based on compounds within the same chemical space as the training set data, suggested that the model performances were roughly similar. The MAE, Kendall τ and S(0) values were comparable in all models, including the three ANNE models. Examination of the SCI revealed three groups of values: RF, ANNE AZ, SVM, MLR and KPLS all had SCI values greater than 0.9; PLS and ANNE had SCI values between 0.7 and 0.9 while ANNE Random had a negative SCI value. Again, these results were explained by examination of the SALI curves (Figure 3b). It is clear that the different models predicted the order across the SALI edges with varying degrees of accuracy, depending on the X value. Of particular note is the observation that ANNE Random again had a negative S(X) value, crossing S(X) = 0 at an X of 0.2; thus, over most of the prediction space this model mispredicted the rank ordering.

Based on these results, there was no *a priori *link between the training and test set values. This may be somewhat expected, since the training sets predicted themselves, whereas the test sets represented unknowns. Thus, the training set performance would present a biased view of the model performance.

Also note that the S(0) and Kendall τ values presented essentially the same measure of the model's ability to properly rank order compounds (S(0) vs. Kendall τ, slope = 0.98, R2 = 0.97, data not shown). Kendall τ, by definition, measures the strength of the relationship between two variables [15]. In addition, since the interpretation of Kendall's τ in terms of the probabilities of observing the agreeable (concordant) and non agreeable (discordant) pairs is very direct, S(0) would yield similar results if it also has that property.

#### Prospective sets

**Figure 4:**
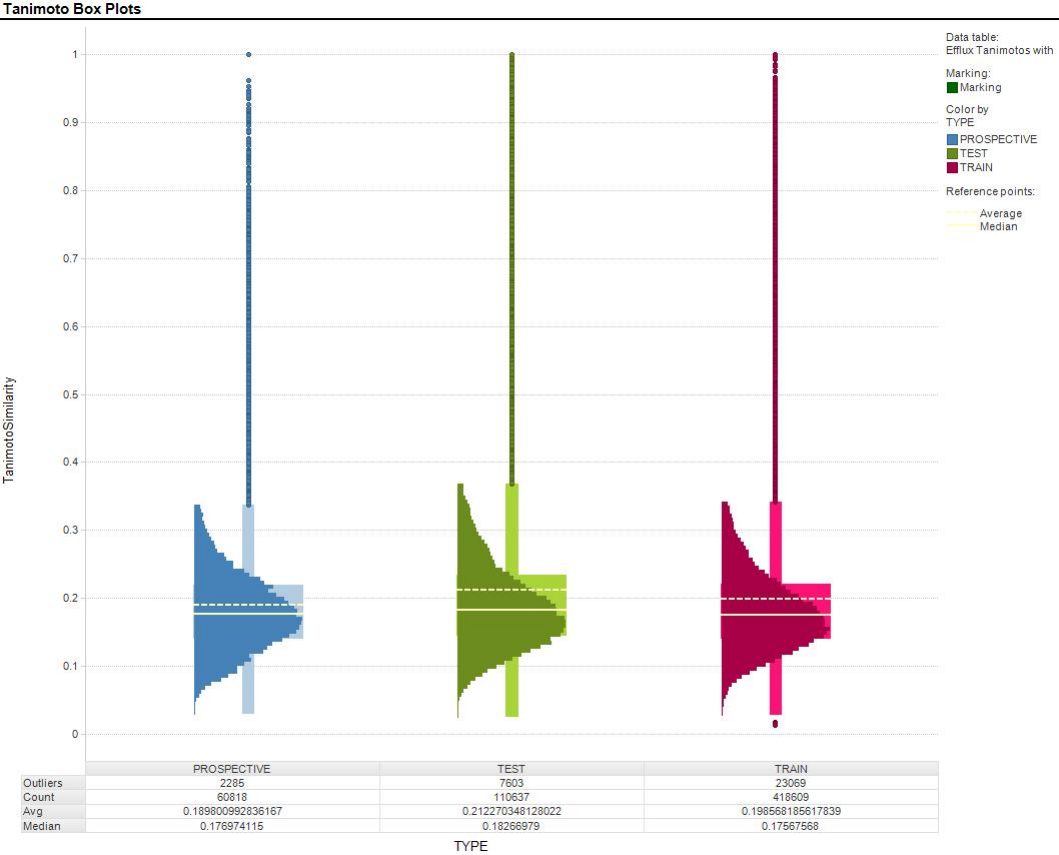
**Box Plots of Daylight FP Scores of Training, Test and Prospective Sets**. Solid line, median Tanimoto score; dashed line, average Tanimoto score. Box shows Q1-Q3. Dots are outliers (greater than upper quartile + 1.5 times interquartile range)

In order to better assess the model quality, a prospective set of compounds which were not in the chemical space (that is, they were marketed drugs and drugs being developed within AstraZeneca for different targets and based on different scaffolds than the compounds used in the training set) of the training/test set compounds was tested in the MDR1-MDCK assay, and was also evaluated in each of the predictive models. Though these compounds were not in the same chemical space as those used in the training and test sets, their Tanimoto distances from the training set were similar to the Tanimoto distances of the test set compounds (Figure 4). Because the RF model as implemented with AstraZeneca does no t identify prediction outliers, it was not used in this evaluation. With the prospective set, the Kendall τ and S(0) values were generally around 0.35 with the exception of MLR, where the values were approximately 0.15. Thus, the models' abilities to rank order compounds overall were moderate, with the exception of MLR which was poor. The MAE values, on the other hand, showed larger differences. KPLS had the lowest MAE value of 0.19, suggesting that on average the KPLS will accurately predict the efflux value within a factor of 5% for this data set. MLR mean prediction error (0.52) was within a factor of 3, while the remaining models had mean errors (~0.3) of approximately a factor of 2. Efforts to correlate training set SCI values with any of the prospective set quality measurements were unsuccessful (data not shown).

As with the training and test set results, the SCI values told a different story (Figure 3c). Here, ANNE, SVM, PLS and ANNE Random were very good at accurately ordering compound pairs across nodes. MLR, KPLS and ANNE AZ had similar albeit somewhat lower ability to correctly order compounds. Again, examination of the SALI curves explained the SCI values. At all X values larger than approximately 0.1, the four best models had a prediction ordering accuracy o f 100%. MLR, KPLS and ANNE AZ had S(X) values < 0 between X = 0.1 and 0.25. These data suggested that, for accurately predicting efflux values, KPLS would be the model of choice. O n the other hand, to more accurately rank order compounds, ANNE, SVM, PLS and ANNE Random would be the better choices.

### Comparison of ADMET Predictor, in house Random Forest and SIMCA PLUS PLS using 3D descriptors

#### Training sets

The results of these studies are summarized in Table 2 All models with the exception of RF had MAE values of approximately 0.2, and Kendall τ and S(0) values approximating 0.6. Similar to the results with 2D descriptors, the RF model had a lower MAE and higher Kendall τ and S(0) values.

**Table 2:** Summary of 3D model performance

Model	ANNE	SVM	MLR	KPLS	RF	PLS
**Training set**						

MAE	0.19	0.21	0.20	0.22	0.10	0.22

Kendall	0.64	0.62	0.62	0.58	0.86	0.60

SCI	0.67	0.73	0.87	0.86	0.99	0.13

S(0)	0.64	0.62	0.62	0.58	0.86	0.59

S(1)	1.00	1.00	1.00	1.00	1.00	-1.00



**Test Set**						

MAE	0.20	0.22	0.23	0.25	0.20	0.23

Kendall	0.55	0.52	0.49	0.43	0.56	0.48

SCI	0.93	0.83	0.83	-0.74	-0.66	0.73

S(0)	0.56	0.52	0.50	0.44	0.57	0.49

S(1)	1.00	1.00	-1.00	-1.00	-1.00	1.00


**Prospective Set**						

MAE	0.35	0.34	0.74	0.34	*	0.32

Kendall	0.34	0.35	-0.09	0.38	*	0.34

SCI	-0.65	-0.49	-0.90	0.80	*	-0.69

S(0)	0.35	0.36	-0.07	0.39	*	0.35

S(1)	-1.00	-1.00	-1.00	1.00	*	-1.00

Models using ADMET 3D Predictor descriptors and Kohonen map: ANNE, ADMET Predictor neural net; SVM, ADMET Predictor support vector machine; MLR, ADMET Predictor multiple linear regression; KPLS, ADMET Predictor kernel partial least squares; RF, Pipeline Pilot random forest; PLS, SIMCA-P+ partial least squares. The performance properties of the models were calculated as described in CALCULATIONS AND STATISTICS. The properties were not calculated for RF since prediction outliers could not be identified

**Figure 5:**
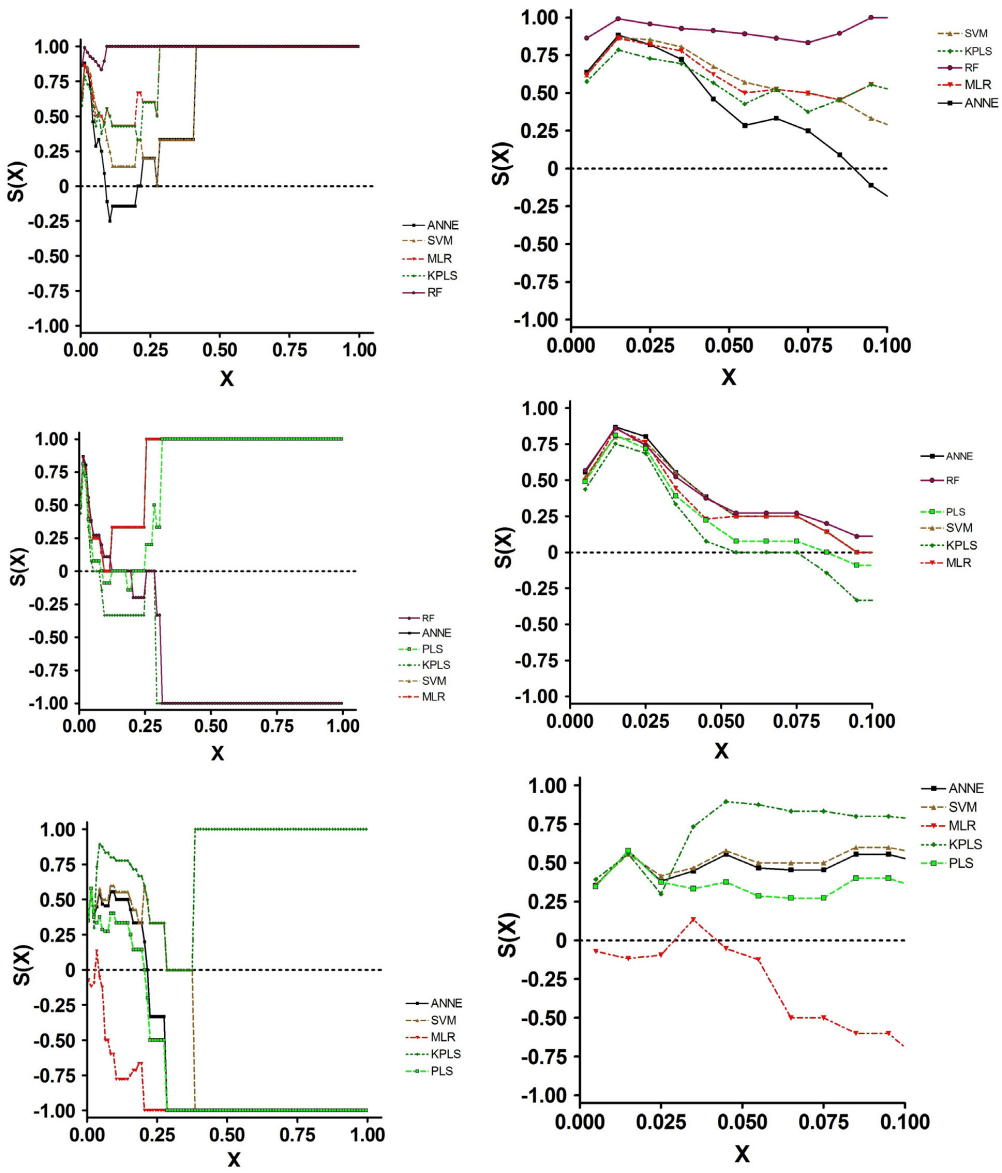
**SALI Curves for Efflux Prediction Data Generated using 3D Descriptors**. 1a, Training Set; 1b, Test Set; 1c, Prospective Set.

Examination of the SCI values shows that RF had a value of 0.99, suggesting that this model was excellent at predicting edges. All other models had SCI values between 0.65 and 0.9 with the exception of PLS with a value of 0.13. Examination of the SALI curves (Figure 5a) reveals a qualitative difference in the curves models tested 2D vs. 3D descriptors. Whereas the curves from models with 2D descriptors differed across the entire range of X values, the models based on 3D descriptors showed large differences at X values between 0 and 0.4, then reached S(X) = 1 at larger values of X. This suggests that the ADMET Predictor 3D descriptors significantly improve the predictability of models across the entire range of X, particularly at large SALI values and in the case of efflux, should be included when possible.

#### Test sets

Based on MAE, all models were generally similar, with KPLS showing a slightly greater inaccuracy. Kendall τ values suggested that ANNE and RF had slightly better performance, while KPLS performance was somewhat less optimal. As with all of the data sets, the S(0) values agreed with the Kendall τ values. In contrast to the statistical and S(0) values, the SCI results showed a wide range of values. Thus, ANNE, SVM and MLR appear to be superior to all of the other models; PLS had a somewhat lower quality, while RF and K PLS were poor in their quality. Examination of the SALI curves (Figure 5b) demonstrated the reason for the differences in model quality. All models reached S(X) = 0 at an X of approximately 0.1. ANNE, SVM and MLR increased to S(X) = 1 as X increased to 0.25, while PLS S(X) reached 1 at X = 0.35. However, at an X of 0.15, both RF and KPLS were at or below 0 (random ordering of compound pairs across the SALI edges) and attained -1 by X = 0.3. Thus, over most of the SAL I space, both RF and KPLS mispredicted the order across the edges.

#### Prospective sets

The same prospective compound set used to evaluate the 2D model performance was used as a benchmark for the 3D models. These results indicate that all models with the exception of MLR had comparable performance base d on MAE, Kendall τ and S(0) values. In fact, these values were almost identical across the models. In contrast, MLR had markedly worse performance for these parameters. The MAE value was about twice that of the other models. The SCI curves (Figure 5c) indicated that all models reached an S(X) = 0 at some point; only KPLS returned to positive S(X) values and reached 1 at X = 0.4. All other models had negative S(X) values across most of the X range. These results indicate that only KPLS was capable of correctly rank ordering compounds in this prospective data set; the other models mispredicted the order.

Standard approaches to the evaluation of prediction model quality entail the examination of overall statistical properties, such as RMSE, MAE and Kendall τ. While these approaches will provide an overall view of how the model may perform across the entire span of chemical space, they do little to define the quality of the model within the predictive space being examined. As a consequence, one's decision regarding the best model to use will be based on incomplete information about how and where best to use the model. In particular, one will have no knowledge as to whether the model works only in the "linear" range of the prediction space or will be able to effectively extrapolate out to more "nonlinear" regions when activity cliffs are present. Using the SALI approach, one obtains both a qualitative (graphical) and quantitative (SCI, S(0), S(1)) assessment of the model performance enabling better utilization and confidence in the predictive power of the models.

Based on standard statistical measures (MAE, Kendall τ), the performance of various prediction approaches (with the exception of RF) on the training sets with 2D descriptors was largely similar. Thus, to predict exact values, any of the models would suffice. However, The SCI revealed marked differences in performance when the goal is rank ordering of compound pairs, a very useful comparison when predicting in novel chemical space to instruct drug discovery. Because MLR and RF showed very high SCI values, either of these two models would be expected to be useful for predicting efflux. At the other extreme, the neural net model built using a random selection method for the training and test sets would not be expected to be useful, since this model was unable to accurately rank order compounds across the SALI edges. In addition, these results suggest that the selection of the training and test sets significantly impacts the quality of the resultant prediction model as would be anticipated from general predictive modeling experience.

When evaluating prediction models, test set data are typically used to assess model performance with unknowns. The test set standard statistical results had similar though slightly lower quality results that those ob served with the training sets. Based on these results, choosing which model performed better would be difficult, since all seem to have similar prediction accuracy. However, the SCI clearly differentiates the model performance. ANNE Random is expected to be significantly inferior to all other models, in agreement with the results seen with the training sets. ANNE and PLS, while acceptable, are of lower quality than the remaining models. Based on the combined training and test set results, it can be concluded that MLR and RF would be expected to perform best within this prediction space.

One weakness in the particular data used for model building was that the training set and test set compounds generally came from the same chemical series. Ideally one would want compounds from outside of the chemical space than that used to build the model to better assess model performance. In an effort to address this concern, compounds from different chemical series were chosen as a prospective test set. In this case, exclusion of compounds which were identified by the prediction software as outside of the prediction space was found to result in an improvement in the calculated model performance values, indicating that the prediction software accurately identified the limits of its predictive ranges. With the 2D descriptors, the standard statistical measures did demonstrate differences in model performance. KPLS had the best overall accuracy while MLR had the worst with all others being similar. The Kendall τ and S(0) values were approximately 0.35 for all models except MLR, where they were about 0.15. However, this was not the case with rank ordering. Neither KPLS, MLR nor neural nets with AZ descriptors was effective at rank ordering compounds while neural nets with ADMET descriptors, SVM and PLS were effective.

In agreement with previous studies, the selection of training set and test set compounds used for the model building is critical [16]. *SimulationsPlus *used Kohonen mapping to select the training and test sets, a methodology which seeks to effectively map the property space delineated by the proscribed descriptors. This approach was found to be more effective than randomly selecting the training and test sets. This was evident from the difference in SCI and S(1) values observed with the neural net models built using *ADMET Predictor *using the Kohonen map vs. random selection. The model with randomly selected sets generated the lowest values in both the training set, where model performance should be "the best" and in the test set.

Generally, there was not a large difference in model performance for prediction of efflux in the prospective data set when using 2D vs. 3D descriptors as determined by MAE, Kendall τ, or S(0). There was, however, a large difference in the SCI values in the training sets. The training set results, though they predict themselves, provide a picture of the best possible outcomes. The observation that the SCI values in the training sets using 3D descriptors for efflux when building prediction models are larger than those built using 2D descriptors suggests that the 3 D descriptors will allow more accurate mapping of the SALI landscape, which should translate into higher quality models.

It is important to point out that the SALI range for the data sets varied according to the particular data set. The maximum SALI value for the training, test, and prospective data sets were approximately 200, 200 and 75, respectively, even though the prospective set had a slightly greater spread of Tanimoto values (Figure 4) Thus, the SALI summary values (SCI, S(0), S(1)) represent the SALI landscape covered by that data set only. This is very important in model evaluation especially in the context of one project or chemical series. However, it would be less useful for generalizing the model quality over the entire SALI landscape covered by the training set since one is only looking at a portion of the entire prediction range and may be presenting a biased view of the model quality. A better representation of the model performance overall is provided by an appropriately chosen test set which more accurately reflects the SALI landscape.

Calculation and evaluation of SALI and SCI values could be incorporated into any model building paradigm. One such approach to the use of these parameters would be as follows. Various prediction models would be built using the desired descriptors, training sets, and calculation engines. The SALI values for the resultant test and training sets should be calculated, and the models with the largest SCI, S(0), and S(1) values would be chosen for continued evaluation and implementation. As one generates measured and predicted data on compounds within a project, one would use the SALI approach to continuously monitor the performance of the model for predicting the desired property. When the SCI, S(0), and/or S(1) values fall below some critical threshold, the model should be updated with the new data to improve the model performance. This will allow one to continually have knowledge of th e applicability of the model in the desired chemical space.

There is still limited knowledge of the applicability of SCI to prediction model evaluation. The limitations of this approach have been summarized previously (Guha and Van Drie [9]). First and foremost is the requirement for a property driven by specific molecular interactions. Also, standard physical chemical properties (solubility, logD) are not readily amenable to SCI evaluation as they are not likely to have activity cliffs. Finally, more work needs to be done to identify the optimal comparison paradigm for assessment of SALI. In the present report, the SALI and SCI values were all internal to the individual data set used, resulting in different maximal SALI values. The predictive power of this approach may be improved by comparing the performance of the test and prospective sets with those of the training sets. This would likely result in the same maximum SALI value for all data sets allowing more direct comparison s of model performance with the various data sets.

## 5. Conclusions

Regulatory and competitive changes in drug discovery are driving an increase in the use of predictive sciences to speed up the development process, reduce costs, and improve safety. Though hardware and software improvements have facilitated and spread the use of prediction models, methodologies for evaluation of the model performance have not kept pace. The recent publications by Guha and Van Drie [8,9] have presented a novel approach to model performance evaluation in the context of SAR.

We have applied the SALI approach to evaluate several models for predicting efflux in MDR1-MDCK cells. The results presented here support the utility of this approach in the evaluation of model performance. Several observations here were identified as being important. First, use of SALI identified models which were better at predicting the relative order of compounds across SALI edges for small and large SALI values. This information, coupled with the SALI curves, allows evaluation of the utility of the model for correctly identifying large activity cliffs, a common occurrence in biochemical SARs. This contrasts to standard statistical approaches which will merely produce one overall number that does not discriminate large and small activity cliffs and, therefore, provides no guidance in model performance for 'nonlinear' processes.

Second, the aggregate SCI value was observed to be different from and complementary to accuracy measures such as MAE. Thus, one could potentially end up with models that would more accurately predict values, but would not necessarily do as good a job at identifying activity cliffs or at correctly ordering compounds across SALI edges. While the former property is certainly valuable, the latter property would be more generally utile when expanding into unknown chemical space where knowledge of the absolute value of a property may be less important than knowledge of "is this better or worse".

The use of structure-activity landscape indices (SALI) and the SALI curve integral (SCI) was found to be very powerful in the evaluation of performance models, particularly with respect to rank ordering of compound pairs. In particular, this approach allows one to evaluate a model's utility in the detection of large activity cliffs, a common occurrence within drug discovery. It is the recommendation of these authors that this approach be incorporated as a quantitative and qualitative step in the evaluation of prediction models.

## 6. Abbreviations

SALI: Structure-Activity Landscape Index; SCI: SALI Curve Integral; ANNE: neural net; SVM: support vector machine; KPLS: kernel partial least squares; PLS: partial least squares; RF: random forest; MDR1-MDCK: multi-drug resistance gene 1 transfected Madine-Darby canine kidney cells; MAE: mean absolute error.

## 7. Competing interests

The authors have no competing interests.

## 8. Authors' contributions

NCLJr collated the data, performed all calculations and results interpretations, guided the design of the VBA calculation engine; KR designed and built the VBA calculation engine; JB reviewed all efflux experimental data to verify experimental integrity; LT designed, wrote and validated the robotics procedures which enabled the execution of the efflux experiments. All authors have read and approved the final manuscript.

## References

[B1] Kapetanovic IM Computer-aided drug discovery and development (CADDD): in silico-chemical-biological approach Chem. Biol. Interact 2008 171 2 165 176 10.1016/j.cbi.2006.12.006 17229415 PMC2253724

[B2] Dutton G In Silico tools streamline drug design GEN 2006 26 4

[B3] Hou T Want J Zhang W Wang W Xu X Recent advances in computational prediciton of drug absorption and permeability in drug discovery Curr. Med. Chem 2006 13 2653 2667 10.2174/092986706778201558 17017917

[B4] Van de Waterbeemd H Gifford E ADMET in silico modeling: Towards prediction paradise? Nature Reviews 2003 2 192 204 10.1038/nrd1032 12612645

[B5] Penzotti JE Landrum GA Putta S Building predictive ADMET models for early decisions in drug discovery Curr. Opin. Drug Disc. Dev 2004 7 49 61 14982148

[B6] Poggesi I Predicting human pharmacokinetics from preclinical data Curr. Opin. Drug Disc. Dev 2004 7 100 111 14982153

[B7] Egan WJ Zlokarnik G Grootenhuis PDJ In silico prediction of drug safety: Despite progress there is abundant room for improvement Drug Disc. Today: Tech 2004 1 381 387 10.1016/j.ddtec.2004.11.002 24981618

[B8] Guha R Van Drie JH Assessing how well a modeling protocol captures a Structure-Activity Landscape J Chem Inf Model 2008 48 1716 1728 10.1021/ci8001414 18686944

[B9] Guha R Van Drie J The Structure-Activity Landscape Index: Indentifying and quantifying activity cliffs J. Chem. Inf. Model 2008 48 646 658 10.1021/ci7004093 18303878

[B10] Morgan ET Goralski KB Piquette-Miller M Renton KW Robertson GR Chaluvadi MR Charles KA Clarke SJ Kacevska M Liddle C Richardson TA Sharma R Sinal CJ Regulation of drug-metabolizing enzymes and transporters in infection, inflammation, and cancer Drug. Met. Disp 2008 36 205 216 10.1124/dmd.107.018747 18218849

[B11] Mizuno N Niwa T Yotsumoto Y Sugiyama Y Impact of drug transporter studies on drug discovery and development Pharm. Reviews 2003 55 425 461 10.1124/pr.55.3.1 12869659

[B12] Seelig A A general pattern for substrate recognition by P-glycoprotein Eur. J. Biochem 1998 251 252 261 10.1046/j.1432-1327.1998.2510252.x 9492291

[B13] Kohonen T Analysis of a simple self-organizing process Biol. Cybernetics 1982 44 135 140 10.1007/BF00317973

[B14] Kohonen T Self-organizing formation of topologically correct feature maps Biol. Cybernetics 1982 43 59 69 10.1007/BF00337288

[B15] Conover WJ Practical Non-Parametric Statistics 1980 2 John Wiley and Sons, New York

[B16] Guha R Serra JR Jurs PC Generation of QSAR sets with a self-organizing map J. Mol. Graph. Model 2004 23 1 14 10.1016/j.jmgm.2004.03.003 15331049

